# Efgartigimod in the treatment of immune checkpoint inhibitor-related myasthenia gravis -myositis overlap syndrome: a case report

**DOI:** 10.3389/fimmu.2026.1818836

**Published:** 2026-07-15

**Authors:** Ke Li, Juanjuan Hu, Zhiji Gan, Jiao Chen, Ye Tian, Ruiqing Luo, Xueliang Qi

**Affiliations:** Department of Neurology, The Second Affiliated Hospital of Nanchang University, Nanchang, China

**Keywords:** efgartigimod, immune checkpoint inhibitor, myasthenia gravis, myositis, treatment

## Abstract

**Background:**

A subset of cancer patients receiving monoclonal antibody PD-1/PD-L1 inhibitors may develop immune checkpoint inhibitor (ICI)-related neurological complications, such as ICI-related myasthenia gravis(MG)-myositis overlap syndrome and ICI-related myocarditis. Standard management typically involves intravenous immunoglobulin (IVIG), plasma exchange (PE), and high-dose corticosteroids. The use of efgartigimod, a neonatal Fc receptor blocker, for the treatment of ICI-related MG-myositis overlap syndrome remains investigational, with only four relevant cases all representing overlap syndromes (MG with myositis, with or without myocarditis) — reported to date.

**Objective:**

This report examines the therapeutic effect of efgartigimod in a patient with cervical cancer who developed ICI-related MG-myositis overlap syndrome after receiving tislelizumab, an anti-PD-1 antibody.

**Methods:**

Clinical data from the patient was collected. Relevant laboratory tests and examinations were conducted. Efgartigimod treatment was administered, and clinical severity was evaluated using standardized assessment scales.

**Results:**

A 69-year-old female with cervical cancer developed bilateral ptosis, dysarthria, and limb weakness following tislelizumab therapy. Examination revealed asymmetric ptosis, weak eye closure, and positive fatigability test. Serum creatine kinase was markedly elevated (553.82 U/L); electromyography showed myopathic changes with fibrillation potentials, while repetitive nerve stimulation was negative. Neostigmine test was positive, and anti-acetylcholine receptor antibodies were detected. She was diagnosed with ICI-related MG-myositis overlap syndrome. After four infusions of efgartigimod (10 mg/kg), creatine kinase normalized, the Activities of Daily Living (ADL) score decreased from 10 to 1, and the Quantitative Myasthenia Gravis (QMG) score improved from 16 to 5. The patient was asymptomatic at the 5 months follow-up with no need for a second treatment cycle.

**Conclusion:**

Efgartigimod produced a positive therapeutic effect in this case with ICI-related MG-myositis overlap syndrome. The therapy was well-tolerated, and no adverse events were reported.

## 1 Introduction

Immune checkpoint inhibitors (ICIs) have become a cornerstone of cancer therapy but can induce immune-related adverse events (irAEs), including rare but severe neurological complications such as ICI-related MG-myositis overlap syndrome. ICI-associated MG-myositis overlap syndrome, and myocarditis are increasingly recognized as part of an overlapping clinical spectrum rather than as strictly separate entities. Among neurological irAEs, ICI-related MG-myositis overlap syndrome represents one of the most frequent and therapeutically challenging syndromes, with myocarditis presenting as an additional severe complication in a subset of patients (1). This overlap poses diagnostic difficulties, as classic myasthenia gravis features such as diurnal fluctuation and decremental responses on repetitive nerve stimulation may be absent. Moreover, the management of this overlap syndrome requires careful balancing of immunosuppression against the need to preserve anti-tumor immunity. Standard management typically involves high-dose corticosteroids, intravenous immunoglobulin (IVIG), or plasma exchange. However, some cases prove refractory to these treatments, highlighting the need for alternative strategies. Efgartigimod, a neonatal Fc receptor blocker that reduces pathogenic IgG levels, is approved for generalized MG. Its use in the treatment of ICI-related MG-myositis overlap syndrome is largely unexplored, with only four cases reported to date (2–5). We describe a patient with cervical cancer who developed concurrent ICI-related MG-myositis overlap syndrome following tislelizumab therapy and was successfully treated with efgartigimod.

## 2 Case description

**Figure 1:**
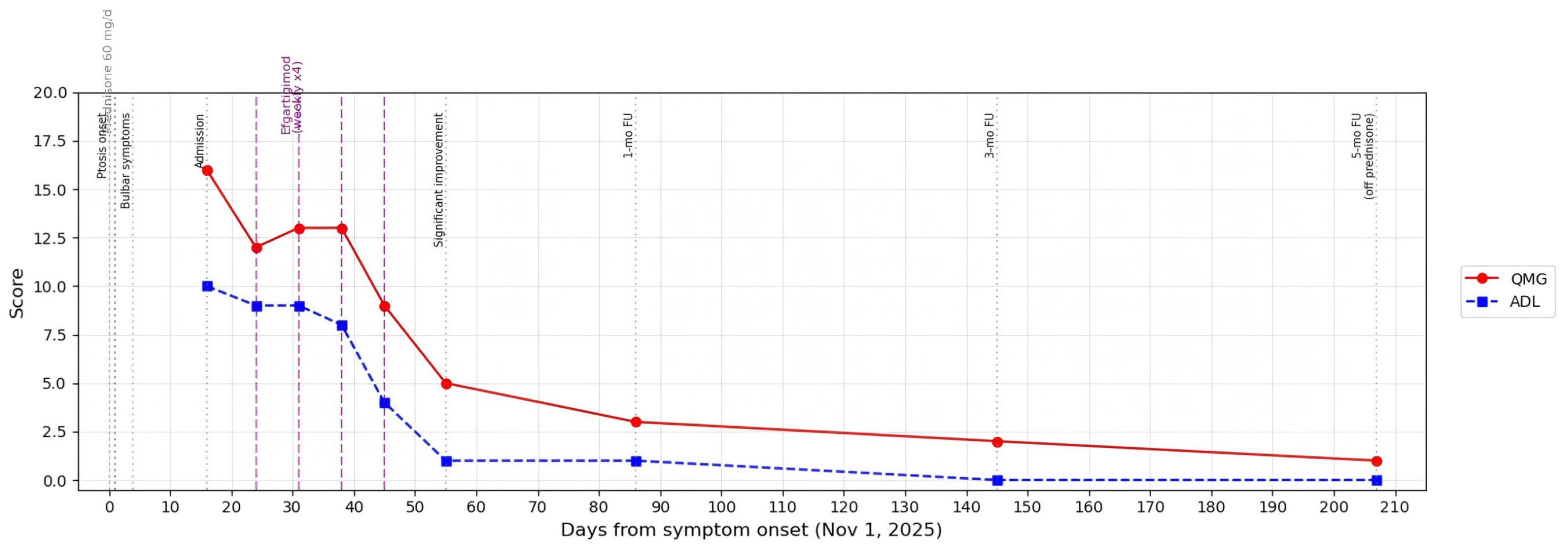
Clinical course, therapeutic interventions, and scoring trends in a patient with ICI−associated myasthenia gravis−myositis overlap syndrome treated with efgartigimod. Serial changes in Quantitative Myasthenia Gravis (QMG) score (red solid line) and Activities of Daily Living (ADL) score (blue dashed line) are shown from symptom onset (Day 0 = November 1, 2025) through 5−month follow−up. Vertical dashed lines indicate key therapeutic interventions, including initiation of oral prednisone (60 mg/day) and four weekly efgartigimod infusions (800 mg each, Days 24–45). Additional time points denote symptom onset, bulbar worsening, hospital admission, significant clinical improvement, and follow−up visits. The patient showed progressive improvement in both scores, with sustained benefit at 5−month follow−up, at which time prednisone had been discontinued and the patient remained asymptomatic. QMG, Quantitative Myasthenia Gravis; ADL, Activities of Daily Living; FU, follow−up; ICI, immune checkpoint inhibitor.

A 69-year-old female with a known history of grade 2 (high-risk) hypertension, post-stroke sequelae (mild numbness and weakness of the left limbs), and hepatic insufficiency was admitted on November 17, 2025, due to bilateral eyelid ptosis persisting for half a month. She had been diagnosed with invasive squamous cell carcinoma of the cervix on September 24, 2025.Subsequently, on September 26, 2025, she received her first cycle of combination therapy, which included liposomal paclitaxel (270 mg intravenous (IV) infusion, on day 1), carboplatin (550 mg IV, on day 1), and the anti-PD-1 monoclonal antibody tislelizumab (200 mg IV, on day 1). This initial treatment was well-tolerated and did not cause significant adverse effects. On October 21, 2025, she received a second cycle of the same regimen, with carboplatin reduced to 500 mg. Following this cycle, the patient developed nausea, vomiting, poor appetite, and alopecia. She also reported numbness and weakness in her left limbs, but did not experience facial deviation, slurred speech, dizziness, or headache. Ten days after completing the second cycle of PD-1 inhibitor therapy (tislelizumab) on November 1, she developed ptosis. These symptoms did not fluctuate significantly or worsen in the evening, and were not associated with blurred or double vision. The patient sought care at a local hospital, where a neostigmine test was positive. Laboratory evaluation revealed an erythrocyte Sedimentation Rate (ESR) of 104 mm/h. Liver function tests (LFTs) showed elevated alanine aminotransferase (ALT: 161 U/L; normal range 0–35 U/L) and aspartate aminotransferase (AST: 205 U/L; normal range 14–36 U/L). On November 10, 2025, a panel testing for myasthenia gravis autoantibodies (Damy Biotchenology, transfected Cell-Based Assay - CBA) which included AChR, MuSK, LRP4, titin, and RyR antibodies was positive only for anti-titin antibodies (titer 1:32). Treatment was initiated with oral pyridostigmine bromide (60 mg three times daily), prednisone acetate (60 mg once daily), levamlodipine besylate (5 mg once daily), and indobufen (0.2 g twice daily). Starting on November 5, she developed dysarthria characterized by a lowered pitch and throat discomfort. She experienced recurrent coughing when consuming food or liquids more quickly. For further evaluation and management, she was admitted to the hospital on November 17, 2025. On admission, her blood pressure was 159/93 mmHg (1 mmHg = 0.133 kPa). The patient was conscious, exhibiting fluent but slurred speech, with intact higher cortical functions. Bilateral ptosis was observed, with complete drooping of the right eyelid and partial ptosis of the left eyelid (3:00-9:00 direction). Extraocular movements were complete in all directions of gaze. Pupils were equal in size, round, and reactive to light bilaterally. Eye closure was weak, more pronounced on the right. Functions such as cheek puffing, mastication, swallowing, head elevation, and neck flexion were largely preserved. Gross hearing was normal. Pain and light touch sensation were slightly decreased in the left limbs. Muscle strength in all four limbs was grade V-, with impaired squat-to-stand ability requiring upper limb assistance. Muscle tone was within normal limits, with no evidence of muscle atrophy or hypertrophy. Pathological reflexes were absent bilaterally. Fatigability was most evident in the bilateral orbicularis oculi — sustained upward gaze for 60 seconds progressively worsened ptosis. Limb fatigability was also present: whereas squat-to-stand was already impaired at baseline and required upper-limb assistance, repeated maneuvers could be performed only 2–3 times before the patient was unable to continue. At admission, the QMG score was 16, and the ADL score was 10.On November 17, 2025, laboratory tests revealed significant elevations in muscle enzymes and cardiac biomarkers, including serum creatine kinase (CK: 553.82 U/L; normal range 40–200 U/L), lactate dehydrogenase (LDH: 432.15 U/L; normal range 120–250 U/L), CK-MB (52.66 ng/mL; normal range 0-3.63 ng/mL), myoglobin (Mb: 121.5 ng/mL, normal range 0–58 ng/mL), while troponin I was normal (0.01 ng/mL; normal range 0-0.028 ng/mL). Electrocardiography demonstrated sinus rhythm with atrial premature beats and T-wave changes. Echocardiography (ECG) revealed trace regurgitation of the mitral, tricuspid, and aortic valves, as well as left ventricular diastolic dysfunction (LVDD). Electromyography (EMG) findings showed prolonged insertion potentials with frequent positive sharp waves and fibrillation potentials at rest in the bilateral tibialis anterior, right biceps brachii, and left gastrocnemius muscles. During mild contraction, the right biceps brachii exhibited low-amplitude, short-duration motor unit action potentials that progressed to a full interference pattern with maximal effort. Similar findings were observed in some motor units of the bilateral tibialis anterior muscles during mild contraction. Other examined limb muscles displayed normal motor unit potentials during mild contraction and mixed interference patterns during maximal effort. On the same day, repetitive nerve stimulation (RNS) studies of the trapezius, orbicularis oculi (low frequency), and abductor digiti minimi (low- and high- frequency) muscles revealed no significant decremental or incremental responses. On November 19, 2025, the neostigmine test (1.5 mg intramuscularly) produced clear improvement in ptosis (right eyelid: from complete ptosis to approximately 2–3 mm palpebral fissure; left eyelid: fissure widened from 3:00–9:00 to 2:00–10:00 direction) and eye closure weakness, but limb strength and squat-to-stand ability did not appreciably change. Serological testing on November 22, 2025, repeated testing at Jiangxi Dian Diagnostics using the same CBA panel identified positive anti-AChR antibodies (titer 1:10). Anti-titin antibodies (titer 1:30) were confirmed as positive. Diaphragmatic ultrasound performed on November 24, 2025, revealed reduced movement and a decreased change ratio of the right hemidiaphragm during quiet breathing. Given the patient’s history of monoclonal antibody (ICI) therapy, clinical presentation, and investigative findings, a diagnosis of immune checkpoint inhibitor ICI-related MG-myositis overlap syndrome(Myasthenia Gravis Foundation of America (MGFA) type IIIB) was established. Myocarditis was excluded, based on the repeatedly normal troponin I levels and the fact that the ECG and echocardiographic findings can be fully attributed to the patient’s pre-existing comorbidities. Despite oral prednisone acetate (60 mg once daily) initiated on November 2, 2025, the patient showed no clinical improvement; instead, new bulbar symptoms developed on November 5 and the QMG score reached 16 by admission on November 17.Due to suboptimal response to corticosteroid monotherapy, treatment with the FcRn antagonist efgartigimod was initiated at a dose of 800 mg (10 mg/kg, approximately 800 mg) via IV infusion on November 25, 2025. Within 40 minutes post-infusion, improvement was observed in ptosis and in eye closure weakness: right eyelid ptosis (5:00-7:00 direction) and left ptosis (2:00-10:00 direction) improved, while dysarthria, nasal speech, and coughing with liquids persisted. The QMG and ADL scores were 12 and 9, respectively. Follow-up muscle enzymes on November 30 indicated marked improvement: CK 134.86 U/L, LDH 253.73 U/L, and Mb 172.24 µg/L. The patient received an additional 40 mL infusion of efgartigimod on December 2, 9, and 16. Serial assessments after each infusion yielded ADL scores of 9, 9, 8, and 4 and QMG scores of 12, 13, 13, and 9. Notably, ten days after the fourth infusion, the functional status improved significantly, with an ADL score of 1 and a QMG score of 5. At 1-month follow-up (January 2026), the patient’s ADL score was 1 and QMG score was 3, with serum CK remaining within normal limits. Oral prednisone had been tapered to 20 mg/day without symptom recurrence. At 3-month follow-up (March 2026), the patient remained clinically stable (ADL 0, QMG 2) with normal CK, and prednisone was further reduced to 10 mg/day. At 5-month follow-up (May 2026), the patient was completely asymptomatic (ADL 0, QMG 1) with CK within normal limits, and prednisone had been discontinued. No delayed adverse events related to efgartigimod were observed throughout the follow-up period. (see **Figure 1**).

## 3 Discussion and conclusion

ICIs have significantly advanced cancer therapy by restoring anti-tumor immune responses through blockade of inhibitory receptors on T cells (e.g., CTLA-4, PD-1) or their ligands (e.g., PD-L1) (6, 7). Despite these benefits, their use has led to an increase in immune-related adverse events (irAEs) (8),which can affect nearly all organ systems; neurological irAEs occur in approximately 1–5% of cases (9). ICI-related MG-myositis overlap syndrome is a rare but potentially life-threatening irAE, with an incidence of approximately 0.12–0.2%, and a high mortality rate of 30% to 50% (9–11). It is important to distinguish between exacerbation of pre-existing autoimmune MG triggered by ICI therapy and denovo ICI-related MG-myositis overlap syndrome (with or without myocarditis); the previously reported cases treated with efgartigimod, as well as the present case, all belong to the latter category (12).In the present case, the patient developed ptosis, dysarthria, and limb weakness after receiving tislelizumab, an anti-PD-1 antibody. A diagnosis of ICI-related MG-myositis overlap syndrome was established based on elevated CK levels, myopathic EMG findings, a positive neostigmine test, and positive AChR antibodies (13). This case exhibited typical irAE features — short latency, severe symptoms, and rapid progression — but also several findings that are atypical for classic autoimmune MG yet increasingly recognized in ICI-associated overlap syndromes: minimal symptom fluctuation without classic diurnal variation, and negative repetitive nerve stimulation (RNS), which in this setting may reflect either a false-negative result or an overdiagnosis of MG when myositis alone causes MG-like symptoms, together with the co-occurrence of MG and myositis as part of a multi-system immune-mediated process. These observations align with the study by Plomp et al., which reported that among ICI-myositis patients, fatigability and RNS decrement were each found in only 2%, whereas MG-like oculomotor symptoms were present in 50% (1); therefore, MG may be overdiagnosed in this context, and a negative RNS should be interpreted cautiously, considering both diagnostic possibilities. The aminotransferase elevation in this case was most likely attributable to muscle injury, and independent hepatic irAE was excluded. Nonetheless, the simultaneous occurrence of MG and myositis highlights the potential for ICIs to trigger multi-organ immune-mediated injury. Thus, a comprehensive systemic assessment — including monitoring of cardiac, hepatic, and thyroid function — remains essential for patients receiving ICI therapy who develop a typical irAE.

Treatment of ICI-related MG-myositis overlap syndrome typically involves corticosteroids combined with IVIG or plasma exchange, with combination therapy proving more effective than corticosteroid monotherapy (11). For refractory cases, eculizumab has been reported as an alternative (14, 15).Efgartigimod, an FcRn antagonist, accelerates the degradation of pathogenic IgG autoantibodies (16, 17). Notably, as most ICIs are themselves IgG monoclonal antibodies, efgartigimod could theoretically accelerate their clearance; however, in the acute irAE setting, short-term, cycle-limited use may be clinically justified by simultaneously targeting pathogenic autoantibodies and attenuating ICI-driven toxicity, while avoiding IVIG-associated adverse events and offering a more convenient treatment process (16, 17). Its lack of hepatic CYP450 metabolism also makes it a favorable option for patients with hepatic involvement (18). In our patient, who showed no response to high-dose prednisone (60 mg/day) with a QMG score of 16, efgartigimod was administered. Early improvement in ptosis was noted clinically, though this observation should be interpreted with caution given the drug’s mechanism and possible symptom fluctuation. After one treatment cycle, the QMG score decreased by 11 points and CK normalized, with sustained efficacy at 5-month follow-up, confirming a genuine therapeutic effect. These findings suggest that efgartigimod may effectively control ICI-related MG-myositis overlap syndrome, offering a novel therapeutic option for this complex and severe condition.

Several case reports have demonstrated the efficacy of efgartigimod in severe ICI-related MG-myositis overlap syndrome. The first involved myasthenic crisis (MGFA Class V) with myocarditis and hepatotoxicity; after one cycle, MG-ADL decreased from 11 to 4 and QMG from 26 to 8, with normalization of cardiac and hepatic markers (2). The second described seronegative, steroid-refractory ICI-related MG-myositis overlap syndrome, achieving complete remission after two infusions (3). Cobelas-Cartagena et al. reported the first acute use in pembrolizumab-induced myocarditis, myositis, and myasthenia gravis overlap syndrome (IM3OS), with rapid stabilization of neurological and cardiac parameters (4), extending efgartigimod’s scope to overlap syndromes with myocardial involvement. More recently, Liu et al. described two patients with PD-1 inhibitor–induced MMM overlap syndrome requiring mechanical ventilation, in whom efgartigimod was used as rescue therapy (5). While these two patients showed differing trajectories, such limited observations do not permit conclusions about the influence of antibody phenotype. Moreover, a recent study has questioned the pathogenic role of anti-AChR antibodies in these syndromes, and larger studies are needed (19). Taken together, the reported cases — including ours, with sustained improvement at 5-month follow-up — indicate that efgartigimod may have broad efficacy across the ICI-induced overlap syndrome continuum. Regarding long-term outcomes, a recent study of ICI-related MG-myositis overlap syndrome reported that while acute mortality remains significant, survivors who receive timely and effective treatment may achieve favorable neurological recovery (20). Our patient’s sustained improvement at 5-month follow-up is consistent with this observation, though continued surveillance is warranted.

In summary, ICI-related MG-myositis overlap syndrome is a serious complication of cancer immunotherapy that requires prompt recognition and diagnosis, due to its acute onset and the potential for concurrent myocarditis (21). Efgartigimod, a neonatal FcRn antagonist, rapidly eliminates pathogenic autoantibodies. When combined with corticosteroids, it significantly improves symptoms and offers a safe, effective, and novel therapeutic option for this condition. Future prospective studies are needed to determine the optimal timing, treatment duration, and long-term safety profile of efgartigimod when administered with ICIs in this patient population.

## Data Availability

The original contributions presented in the study are included in the article/supplementary material. Further inquiries can be directed to the corresponding author.
